# The A B C Medical Diary and Visiting List for 1893

**Published:** 1892-12

**Authors:** 


*The A B C Medical Diary and Visiting List for* 1893. London: CHARLES LETTS & CO. and BURROUGHS, WELLCOME & CO.

This list is of the same size as last year's edition, and contains space for recording visits to either 56 or 112 patients, and pages for various other memoranda. With the diary are bound up 132 pages of closely-printed matter, forming what is called the "Excerpta Therapeutica," which consists of condensed information arranged in alphabetical order. We notice that the drugs mentioned are for the most part those of which Messrs. Burroughs, Wellcome & Co. have a special preparation, and we have every reason to consider them worthy of confidence.

